# A 19-Gene expression signature as a predictor of survival in colorectal cancer

**DOI:** 10.1186/s12920-016-0218-1

**Published:** 2016-09-08

**Authors:** Nurul Ainin Abdul Aziz, Norfilza M. Mokhtar, Roslan Harun, Md Manir Hossain Mollah, Isa Mohamed Rose, Ismail Sagap, Azmi Mohd Tamil, Wan Zurinah Wan Ngah, Rahman Jamal

**Affiliations:** 1 UKM Medical Molecular Biology Institute, Universiti Kebangsaan Malaysia (UKM), Cheras, Kuala Lumpur, Malaysia; 2 Department of Physiology, Faculty of Medicine, Universiti Kebangsaan Malaysia, Jalan Yaacob Latif, Bandar Tun Razak, Cheras, 56000 Kuala Lumpur, Malaysia; 3 Histopathology Unit, Department of Pathology, Universiti Kebangsaan Malaysia Medical Centre, Kuala Lumpur, Malaysia; 4 Department of Surgery, Universiti Kebangsaan Malaysia Medical Centre, Kuala Lumpur, Malaysia; 5 Department of Community Health, Faculty of Medicine, Universiti Kebangsaan Malaysia, Kuala Lumpur, Malaysia

**Keywords:** Colorectal cancer, Microarray analysis, Survivalm, Real-time PCR

## Abstract

**Background:**

Histopathological assessment has a low potential to predict clinical outcome in patients with the same stage of colorectal cancer. More specific and sensitive biomarkers to determine patients’ survival are needed. We aimed to determine gene expression signatures as reliable prognostic marker that could predict survival of colorectal cancer patients with Dukes’ B and C.

**Methods:**

We examined microarray gene expression profiles of 78 archived tissues of patients with Dukes’ B and C using the Illumina DASL assay. The gene expression data were analyzed using the GeneSpring software and R programming.

**Results:**

The outliers were detected and replaced with randomly chosen genes from the 90 % confidence interval of the robust mean for each group. We performed three statistical methods (SAM, LIMMA and *t*-test) to identify significant genes. There were 19 significant common genes identified from microarray data that have been permutated 100 times namely *NOTCH2, ITPRIP, FRMD6, GFRA4, OSBPL9, CPXCR1, SORCS2, PDC, C12orf66, SLC38A9, OR10H5, TRIP13, MRPL52, DUSP21, BRCA1, ELTD1, SPG7, LASS6* and *DUOX2*. This 19-gene signature was able to significantly predict the survival of patients with colorectal cancer compared to the conventional Dukes’ classification in both training and test sets (*p* < 0.05). The performance of this signature was further validated as a significant independent predictor of survival using patient cohorts from Australia (*n* = 185), USA (*n* = 114), Denmark (*n* = 37) and Norway (*n* = 95) (*p* < 0.05). Validation using quantitative PCR confirmed similar expression pattern for the six selected genes.

**Conclusion:**

Profiling of these 19 genes may provide a more accurate method to predict survival of patients with colorectal cancer and assist in identifying patients who require more intensive treatment.

**Electronic supplementary material:**

The online version of this article (doi:10.1186/s12920-016-0218-1) contains supplementary material, which is available to authorized users.

## Background

Colorectal cancer is one of the major causes of cancer mortality in both sexes worldwide. The reported number of CRC patients has increased to 1.4 million and associated with 694 000 deaths globally in 2012 [1]. CRC is staged according to the extent whether it has spread through the wall of colon and rectum or to other parts of the body [2]. The prognosis is influenced by the stage of CRC at the time of diagnosis [3]. Based on the National Cancer Institute's Physician Data Query system, the 5-year survival rate for Dukes’ A patients was 80 to 95 %, Dukes’ B 55 to 80 %, Dukes’ C 33 to 55 % and Dukes’ D less than 15 % [4]. These data showed the correlation between survival and staging where the higher stage of CRC patients is associated with a lower survival rate. However, a previous study reported that the survival rate of Dukes’ B patients with high risk pathological factors or low nodes involvement was lower than Dukes’ C patients who had one positive node [3]. Thus, the current staging method needs to be improved to provide a more accurate prognostication for CRC patients. The common practice in managing Dukes’ B patients is a combination of surgery, chemotherapy and/or radiation therapy [5]. Whether this should be applied for all cases is still debatable [3]. The adjuvant chemotherapy may benefit the Dukes’ B patients with high risk features but this is still not routinely recommended. This is due to less benefit obtained from the adjuvant chemotherapy as 10-20 % of patients will develop recurrence [6]. For Dukes’ C patients, the adjuvant chemotherapy became a standard treatment after showing a 40 % reduction of recurrence rate [7]. Another study in 2004 has demonstrated that the overall 5-year survival rate was poor in patients with Stage IIb compared to those with stage IIIa [4]. However, this result may be due to the misclassification of staging which leads to poor survival in untreated patients with micrometastasis [4]. Clearly, there are pitfalls in using the current staging system for prognostication purposes.

Nowadays, the development of high throughput technologies such as RNA sequencing [8, 9] and microarray [10, 11] become popular to generate gene expression profiling in understanding of cancer. Microarray technology is still valuable and promising technology for many years as it is more affordable compared to the RNA sequencing. Eschrich et al. (2005) used cancer tissues from patients with Dukes’ B, C and D, who have been follow-up for at least 36 months. They found a 43-gene signature that categorize patients into good and poor survival with 93 % sensitivity and 84 % specificity [12]. But, a large scale validation could not be performed due to the limitation to make decision for adjuvant treatments [11, 13]. Several studies that analyzed patients with Stage II and III CRC have developed molecular classifiers that could stratify patients into high and low-risk groups [14–16]. However, these studies are still in the research phase were not been translated into clinical practice [17]. Furthermore, some studies have used a small number of samples and lack of validation in independent samples to enhance the power of the gene signatures [18]. Our aim for this study was to determine gene expression signatures that could predict survival of CRC patients with Dukes’ B and C CRC, hoping that a set of gene signatures will be able to classify patients into those with good or poor survival as well as to more accurately targeted therapy.

## Methods

### Clinical samples

This is a retrospective study using 78 formalin-fixed paraffin embedded (FFPE) tissues of patients with Dukes’ B (*n* = 37) and Dukes’ C (*n* = 41) CRC patients diagnosed between January 2002 to December 2007 at the Universiti Kebangsaan Malaysia Medical Centre. These samples comprised of patients who survived less than five years (denoted as the poor survival group) and patients who survived more than five years (good survival group). The samples were anonymised throughout this study. Inclusion criteria included the absence of preoperative chemotherapy or radiotherapy. Information about age, gender, race, histology, family history, organ sites and clinical outcomes were recorded. For each patient, their medical records and follow-up data were carefully reviewed to confirm their clinical outcomes and the cause of death if the patients were deceased. The survival of patients was calculated from December 2012 minus the date of the first surgery for those still alive while for those who did not survive, it included the date of death minus the date of the first surgery.

### RNA extraction

Tissue sections of 4-7 μm in thickness were prepared (>80 % representative), stained with hematoxylin and eosin (H&E) and evaluated by the pathologist in charge. RNA was deparaffinized and extracted using High Pure RNA Paraffin Kit (Roche Applied Science, Mannheim, Germany). Proteinase K was added and homogenization was performed for16 h. All steps followed the manufacturer’s protocol. Samples were then stored at –80 °C until they were used. Quantity and purity of the total RNA was determined by the NanoDrop ND-1000 spectrophotometer (Thermo Fisher Scientific, Waltham, MA). Samples with purity between 1.8 to 2.0 (A260/A280) were selected. Quality assessment of total RNA was done using the Bioanalyzer 2100 RNA 6000 Nano kit (Agilent Technologies, Inc., CA, USA) and samples with RNA Integrity Number (RIN) of more than two were selected for the quantitative PCR.

### cDNA mediated annealing, selection, extension and ligation (DASL) assay

Quantitative PCR analysis was performed as the pre-qualifying step prior to cDNA synthesis using the Corbett Rotor-Gene 6000 thermal cycler (Corbett Life Science, Sydney, Australia). Forward and reverse primers for the housekeeping gene RPL13A were obtained from AITbiotech Singapore. PCR amplification with C_T_ value of 29 cycles or less was used in DASL assay (Illumina, San Diego, CA, USA). The assay was conducted according to the manufacturer’s protocol. Raw data files (.idat files) were analyzed using the GenomeStudio software (Illumina, San Diego, CA, USA) to check the data quality control for assessing results of gene expression microarray experiment.

### Microarray analysis

Sample Probe Profile from the GenomeStudio was imported to the third party software called Genespring GX 12.0.2 (Agilent Technologies, Inc., CA, USA). Seventy-eight samples were exploitable with 20793 entities. The data were normalized using quantile algorithm and log-transformed. Baseline transformation of the normalized signal was performed to the median of all samples. Samples were assigned into their survival groups. Hierarchical clustering was performed using Pearson's correlation coefficient and Ward’s criterion.

### Outlier diagnosis for microarray analysis

It is well known that microarray gene-expression data are often contaminated by outliers due to many steps involved in the experimental process from hybridization to image analysis [19, 20]. Most of the popular algorithms for microarray gene-expression data analysis are very much sensitive to outliers [19]. So gene-expression data analysis by these algorithms may produce misleading results in the presence of contaminated observations.

We identified the contaminated observations for each gene using β-weight function [21, 22] and replaced them with the values belonging to the 90 % robust confidence interval (rCI) of the respective group mean. The 90 % rCI for the *j*-th group mean (*μ*_*i*_^(*j*)^) of *i*-th gene is defined by

1 $$ \left[\left({\displaystyle {\widehat{\mu}}_{i,\beta}^{(j)}}-1.644\times {\displaystyle {\widehat{\sigma}}_{i,\beta}^{(j)}}\right./\left.\sqrt{n_j}\right),\left({\displaystyle {\widehat{\mu}}_{i,\beta}^{(j)}}+1.644\times {\displaystyle {\widehat{\sigma}}_{i,\beta}^{(j)}}\right./\left.\sqrt{n_j}\right)\right] $$

where ${\displaystyle {\widehat{\mu}}_{i,\beta}^{(j)}}$ and ${\displaystyle {\widehat{\sigma}}_{i,\beta}^{(j)}}$ are the minimum β-divergence estimators of mean (*μ*_*i*_^(*j*)^) and variance (*σ*_*i*_^2(*j*)^) obtained iteratively as follows

2 $$ {\displaystyle {\mu}_{i,t+1}^{(j)}}=\frac{{\displaystyle {\sum}_{k=1}^{n_j}}{\psi}_{\beta}\left(\left.x\begin{array}{c}\hfill (j)\hfill \\ {}\hfill ik\hfill \end{array}\right|\theta \begin{array}{c}\hfill (j)\hfill \\ {}\hfill i,t\hfill \end{array}\right)x\begin{array}{c}\hfill (j)\hfill \\ {}\hfill ik\hfill \end{array}}{{\displaystyle {\sum}_{k=1}^{n_j}}{\psi}_{\beta}\left(\left.x\begin{array}{c}\hfill (j)\hfill \\ {}\hfill ik\hfill \end{array}\right|\theta \begin{array}{c}\hfill (j)\hfill \\ {}\hfill i,t\hfill \end{array}\right)} $$

and

3 $$ \sigma \begin{array}{c}\hfill 2(j)\hfill \\ {}\hfill i,t+1\hfill \end{array}=\frac{{\displaystyle {\sum}_{k=1}^{n_j}}{\psi}_{\beta}\left(\left.{\displaystyle {x}_{ik}^{(j)}}\right|\theta \begin{array}{c}\hfill (j)\hfill \\ {}\hfill i,t\hfill \end{array}\right){\left(x\begin{array}{c}\hfill (j)\hfill \\ {}\hfill ik\hfill \end{array}-\mu \begin{array}{c}\hfill (j)\hfill \\ {}\hfill i,t\hfill \end{array}\right)}^2}{{\left(\beta +1\right)}^{-1}{\displaystyle {\sum}_{k=1}^{n_j}}{\psi}_{\beta}\left(\left.{\displaystyle {x}_{ik}^{(j)}}\right|\theta \begin{array}{c}\hfill (j)\hfill \\ {}\hfill i,t\hfill \end{array}\right)} $$

where $$ {\displaystyle {\theta}_i^{(j)}}=\left(\mu \begin{array}{c}\hfill (j)\hfill \\ {}\hfill i\hfill \end{array},\sigma \begin{array}{c}\hfill 2(j)\hfill \\ {}\hfill i\hfill \end{array}\right) $$, *x*_*ik*_^(*j*)^ is the *k*-th expression in group-*j* of gene-*i*, *i* = 1,2,….,*N* = 20793; *j* = 1,2; *n*_*1*_ *+ n*_*2*_ = 78, and which is known

4 $$ {\psi}_{\beta}\left(\left.x\begin{array}{c}\hfill (j)\hfill \\ {}\hfill ik\hfill \end{array}\right|\theta \begin{array}{c}\hfill (j)\hfill \\ {}\hfill i\hfill \end{array}\right)= \exp \left\{-\frac{\beta }{2}{\left(\frac{x\begin{array}{c}\hfill (j)\hfill \\ {}\hfill ik\hfill \end{array}-\mu \begin{array}{c}\hfill (j)\hfill \\ {}\hfill i\hfill \end{array}}{\sigma \begin{array}{c}\hfill (j)\hfill \\ {}\hfill i\hfill \end{array}}\right)}^2\right\} $$

as β-weight function that we used for outlier detection as mentioned earlier. This weight function produces weights between 0 and 1 for any observation detected. It produces smaller weights only for contaminated observations. So, in this study we consider an observation (*x*_*ik*_^(*j*)^) as a contaminated observation when

4.1 $$ {\psi}_{\beta}\left(\left.x\begin{array}{c}\hfill (j)\hfill \\ {}\hfill ik\hfill \end{array}\right|\widehat{\theta}\begin{array}{c}\hfill (j)\hfill \\ {}\hfill i,\beta \hfill \end{array}\right)<0.2 $$

and replaced it with a value belonging to the 90 % rCI of mean $$ \left(\mu \begin{array}{c}\hfill (j)\hfill \\ {}\hfill i\hfill \end{array}\right) $$ as the defined eq. (1). The β-estimators as defined in eqs. 2 and 3 are highly robust against outliers [21, 22].

### Detection of differentially expressed (DE) gene

We permutated 100 times from one data the microarray data obtained from78 patients. All patients were divided into two subsets of equal numbers i.e., training and test sets. We used the bootstrapped data with three statistical methods (SAM, LIMMA and *t*-test) to each training and test set to detect significantly DE genes between good and poor survival group.

### Survival analysis

#### Cox proportional hazards model and Elastic net estimation

To estimate the relationship between the survival time and the gene expression levels, we used *n* as a sample of n size and *X*_*1*_*, . . .,X*_*p*_ of *p* genes to denote the gene expression level. The survival data for the *i*th patient denoted by (*T*_*i*_*, δ*_*i*_*, x*_*i1*_*, x*_*i2*_*, . . . x*_*ip*_), where *i* = 1, 2, . . ., *n*,*Ti* is the survival time of *i* patient, *δ*_*i*_ is censoring indicator (0 if alive, 1 death) and *x*_*i*_ = {*x*_*i1*_*, x*_*i2*_*, . . ., x*_*ip*_} is the vector of the gene expression level of *p* genes (covariates). We also used the Cox regression model for the hazard of CRC death at time *t* which is defined by

$$ \begin{array}{l}\lambda (t)=\lambda o(t)\kern0.5em  \exp \left({\beta}_1{X}_1+{\beta}_2{X}_2+\cdot \cdot \cdot \cdot +{\beta}_p{X}_p\right)\hfill \\ {}\kern3.5em ={\lambda}_0(t) \exp \left\{{\beta}^TX\right\},\hfill \end{array} $$

where *λ*_0_*(t)* is an unspecified baseline hazard function, *β = {β*_1_*, β*_2_*,* · · ·,*β*_p_*}* is the vector of regression coefficients and *X* = {*X*_1_, *. . .*,*X*_*p*_} is the vector of gene expression levels with the corresponding sample values of *xi* = {*x*_*i*1_, *. . .*, *x*_*ip*_} for the *i*th sample. The the risk score of patient was calculated from the function:

5 $$ \mathrm{Risk}\ \mathrm{Score}=f(X)={\beta}^TX $$

Based on the available sample data, the Cox’s partial likelihood can be written as

$$ L\left(\beta \right)={\displaystyle \prod_{r\in D}\frac{ \exp \left({\beta}^T{x}_r\right)}{{\displaystyle {\sum}_{j\in {R}_r} \exp \left({\beta}^T{x}_j\right)}}} $$

where *D* is the set of indices of the events (e.g., deaths) and *R*_r_ denotes the set of indices of the individuals at risk at time *t*_r_ − 0. The Elastic Net [23] uses a mixture of the L_1_ (lasso) and L_2_ (ridge regression) penalties. In the Elastic Net, the usual partial log-likelihood is penalized by the L_1_ and L_2_ norms of the regression coefficients with weights *λ*_1_ and *λ*_2_, respectively, i.e.,:

6 $$ {\displaystyle {l\left(\beta \right)}_{penalized}}=l\left(\beta \right)-{\lambda}_1{\displaystyle \sum_{i-1}^p\left|{\beta}_i\right|}-{\lambda}_2{\displaystyle \sum_{i-1}^p{\left({\beta}_i\right)}^2} $$

where *λ*_1_ and *λ*_2_ are tuned by maximizing *l*(*β*), and *l*(*β*) is the cross-validated partial log-likelihood (CVL). LASSO and Ridge regression are described by Eq. (2) with *λ*_1_ or *λ*_2_ non-zero, respectively. The *λ*_1_ + *λ*_2_ Elastic Net involves 2D optimization of the penalties. The penalty parameters were tuned 50 times using different folding of the data for calculating CVL, and the penalty parameters with maximum CVL were selected by *pensim* R package, available at http://cran.r-project.org/web/packages/pensim/index.html.

We performed the Elastic Net [23] using the opt2D function of the “*pensim”* R package to predict the survival of CRC patients from microarray data. Using a 10-fold cross-validation, with 50 starts parallelized to 8 processors using the *opt2D* function, we obtained regression coefficients (β) with the optimal penalty parameter for the penalized Cox model, and calculated the risk score for each patient using eq. (5) where *β*_*i*_ is the estimated regression coefficient of each gene in the training data set and *X*_*i*_ is the Z-transformed expression value of each gene. The estimated regression coefficient of each survival related gene given by Elastic Net in eq. (6) in the training data set was also applied to calculate a risk score for each patient in test data set. The linear risk score with greatest cross-validated partial log-likelihood was selected for validation in the test set. We classified all patients into the 2 groups high and low risk groups using the cut-off value (median risk score) in the training set. Patients were assigned to the "high-risk" group if their risk score was more than or equal to cut-off value of risk score, whereas those with less than the cutoff values were assigned as "low-risk". The patients in high-risk group are expected to have a poor outcome. The statistical significance of the predictions was then assessed by the likelihood ratio test on the Cox proportional hazards model. The probe sets were scaled to z-scores per feature for all datasets. An individual patient (test patient) can be checked to predict whether the patient should receive further treatment or no treatment by the fitted risk score (eq. 5), where *X* = {*X*_1_, *. . .*,*X*_*p*_} takes the expression values of selected *p =* 19 genes from the test patient in the real life daily practice.

The values of specificity and sensitivity of the 19-genes was calculated based on the analysis of gene expression from this study as compared to the selected genes from other publications [14, 15].

### Independent external validation

We compared our microarray data with the published datasets obtained from Stage II and III CRC patients from four separate international studies (Australia, USA, Denmark and Norway) [11, 14, 15, 24]. The datasets were accessed online from Gene Expression Omnibus (GEO) (GSE14333, GSE17536/GSE17537, GSE31595 and GSE30378). The platform used was Affymetrix HG-U133 Plus2.0. The raw fluorescence intensity data within CEL files were pre-processed with Robust Multichip Average (RMA) algorithm [8], as implemented with R packages from Bioconductor (http://www.bioconductor.org), and the data were log-transformed. Clinical information of the publicly available microarray data sets was obtained from the published articles and websites. In addition, the data were normalized per gene in each data set by transforming the expression of each gene to obtain a mean of 0 and SD of 1 (Z-transformation) for this study.

### Validation using quantitative PCR (qPCR)

Six genes (*FRMD6, SLC38A9, TRIP13, MRPL52, ELTD1* and *ITPRIP*) were randomly selected for the validation of the microarray data. Results were normalized with *RPL13A* gene. The extracted total RNA was converted to cDNA using Verso cDNA Synthesis kit (Thermo Scientific, UK). For qPCR, 25 μl reactions were set up using 12.5 μl of 2X Solaris qPCR Master Mix, 1.25 μl of Solaris Primer/Probe Set (20X), 1 μl of cDNA template and water to make up to total volume 25 μl. The qPCR reactions were performed using the Rotor-Gene 6000 thermal cycler (Corbett Life Science). Cycling program involved one cycle of enzyme activation at 95 °C for 15 min, 40 cycles consist of denaturation at 95 °C for 15 s and annealing/extension at 60 °C for 60 s.

## Results

### Clinical and pathological features

**Fig. 1:**
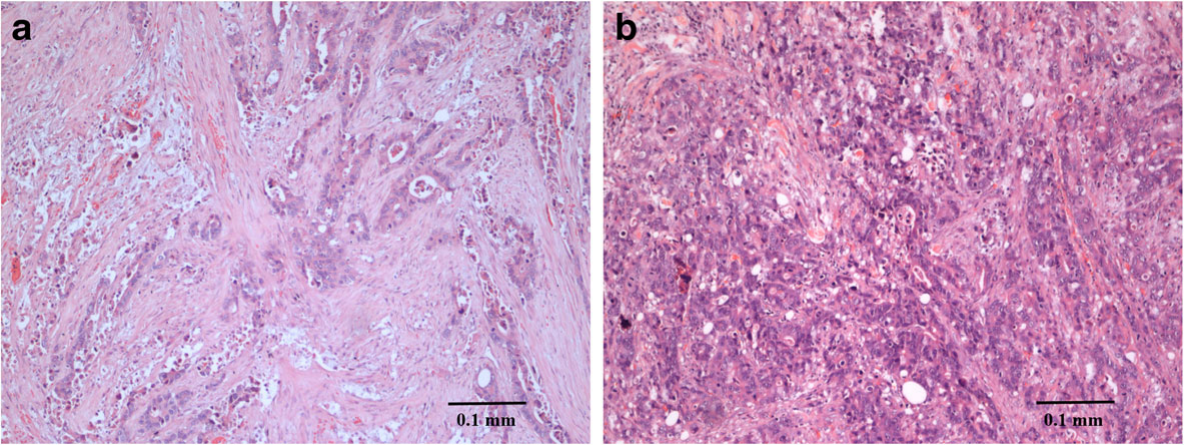
**a** Cancerous tissue section of patients Dukes' B well-differentiated adenocarcinoma. Hematoxylin (purple) stains chromatin in the nucleus and eosin (pink orangish) gives color to the protein that resides in the cytoplasm of muscle cells. Tumor cells appear to thicken and be seen spreading muscular propia but did not penetrate serous layer. **b**. Well differentiated adenocarcinoma Dukes’ C tissue section invaded into muscular propia and involved lymph nodes

Clinical and pathological features of 78 patients were separated into poor and good survival groups of patients who survived less than five years and more than five years respectively. In this study, the 5 year survival rate among patients of Dukes’ B was 59.5 % while Dukes’ C was 36.5 %. It was in concordance to the United State data [4]. The differences in clinical parameters between Dukes’ B and C patients were not statistically significant (Fisher’s exact test *p* = 0.173) (Table 1). Kaplan Meier curves were constructed based on Dukes’ staging and the survival time showed no statistically significant difference (log rank *p* = 0.242, data not shown). Fig. 1 showed the H&E staining results of patient Dukes’ B and C.

**Table 1:** Clinical and pathological features

		Good survival *n* = 39	Poor survival *n* = 39	
		No (%)	No (%)	*p* value
Dukes’	B	22 (56.41)	15 (38.46)	0.173 **
	C	17 (43.59)	24 (61.54)	
Gender	Male	20 (51.28)	20 (51.28)	1.000 **
	Female	19 (48.72)	19 (48.72)	
Age (year)	≤50	4 (10.26)	5 (12.82)	0.235 **
	>50	35 (89.74)	34 (87.18)	
Race	Chinese	29 (74.36)	24 (61.54)	0.226 *
	Malay	9 (23.08)	15 (38.46)	
	Indian	1 (2.56)	0	
Tumor differentiation	Well	26 (66.67)	15 (38.46)	0.051 *
	Moderately	5 (12.82)	15 (38.46)	
	Poorly	1 (2.56)	2 (5.13)	
	Mucinous	2 (5.13)	4 (10.26)	
	No record	5 (12.82)	3 (7.69)	
Clinical outcome	Alive	34 (87.18)	0	0.000 **
	Dead	5 (12.82)	39 (100.00)	
Organ	Colon	25 (64.10)	21 (53.85)	0.357 **
	Rectum	14 (35.90)	18 (46.15)	

* = *p* value was calculated using Pearson Chi-Square

** = *p* value was calculated using Fisher’s Exact Test

[Relevant location: Page 13]

### Identification of DE genes between good and poor survival groups

Based on the eqs. (4 & 4.1), we identified 7.7 % of 20793 probes as contaminated probes (Additional file 1). Then, we updated all contaminated expressions for each gene using the reasonable values belonging to the 90 % rCI of their respective group means as discussed earlier.

Thus, a total of 1500 top-ranked DE genes (using smaller adjusted *p*-values) was selected from each of training and test datasets by each of three statistical tests (See Methods). Overlapping genes obtained by three statistical test were again overlapped between each of the training and test datasets (Additional file 1). Finally we obtained 19 significant DE genes (*NOTCH2, ITPRIP, FRMD6, GFRA4, OSBPL9, CPXCR1, SORCS2, PDC, C12orf66, SLC38A9, OR10H5, TRIP13, MRPL52, DUSP21, BRCA1, ELTD1, SPG7, LASS6 and DUOX*) for further investigation (Table 2).

**Table 2:** Microarray-based changes in gene expression of the 19 genes

Probe ID	Gene symbol	^a^Fold change	Gene name	Expression in poor survival group (Up-regulated/Down-regulated)
7000692	*MRPL52*	-4.32 (-2.59)	mitochondrial ribosomal protein L52	Down-regulated
5700373	*TRIP13*	-3.49 (-3.22)	thyroid hormone receptor interactor 13	Down-regulated
2690324	*ITPRIP*	1.36 (1.23)	inositol 1,4,5-triphosphate receptor interacting protein	Up-regulated
7000184	*SLC38A9*	-3.89 (-3.08)	solute carrier family 38, member 9	Down-regulated
5420070	*FRMD6*	3.65 (2.63)	FERM domain containing 6	Up-regulated
4230739	*SORCS2*	2.96 (3.58)	sortilin-related VPS10 domain containing receptor 2	Up-regulated
6040070	*ELTDI*	3.68 (2.59)	EGF, latrophilin and seven transmembrane domain containing 1	Up-regulated
1190176	*NOTCH2*	3.37 (2.62)	Notch homolog 2	Up-regulated
2570196	*CPXCR1*	-2.62 (-2.18)	CPX chromosome region, candidate 1	Down-regulated
840367	*OR10H5*	-2.20 (-1.93)	olfactory receptor, family 10, subfamily H, member 5	Down-regulated
3450575	*PDC*	-3.06 (-1.75)	phosducin	Down-regulated
2710564	*DUOX2*	2.22 (3.01)	dual oxidase 2	Up-regulated
4560474	*GFRA4*	-2.91 (-1.63)	GDNF family receptor alpha 4	Down-regulated
5690064	*LASS6*	-2.69 (-2.45)	LAG1 homolog, ceramide synthase 6	Down-regulated
3780725	*OSBPL9*	-2.04 (-2.45)	oxysterol binding protein-like 9	Down-regulated
5090025	*C12orf66*	-1.15 (-1.11)	chromosome 12 open reading frame 66	Down-regulated
5870121	*SPG7*	3.64 (4.57)	spastic paraplegia 7 (pure and complicated autosomal recessive)	Up-regulated
620398	*DUSP21*	-3.53 (-2.92)	dual specificity phosphatase 21	Down-regulated
540411	*BRCA1*	-3.88 (-3.07)	breast cancer 1, early onset	Down-regulated

This table shows the probe ID, gene symbols and expression of the 19 genes in the poor survival group compared to the good survival group. ^a^Fold change: training set (test set)

[Relevant location: Page 13]

**Fig. 2:**
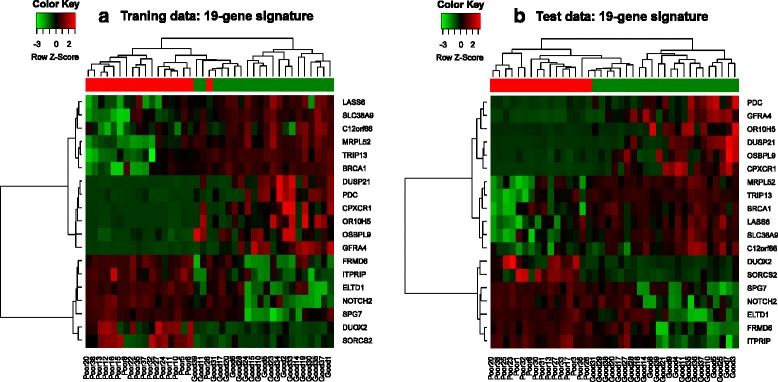
Hierarchical clustering of gene expression datasets. Hierarchical clustering of 78 CRC samples in training and test sets which graphically displays the intensity of the gene expression for each gene. Samples were clustered based on the 19 significant genes. The color of each square boxes represents the ratio of gene expression. Red boxes indicates up regulated genes while green boxes represents down regulated genes. The column represent individual tissue samples while rows represent individual of genes

Figure 2 shows an example of the hierarchical clustering of microarray results based on 19 DE genes from a pair of training set 1 and test set 1.

### Predicting survival of cancer patients from CRC gene expression data

**Fig. 3:**
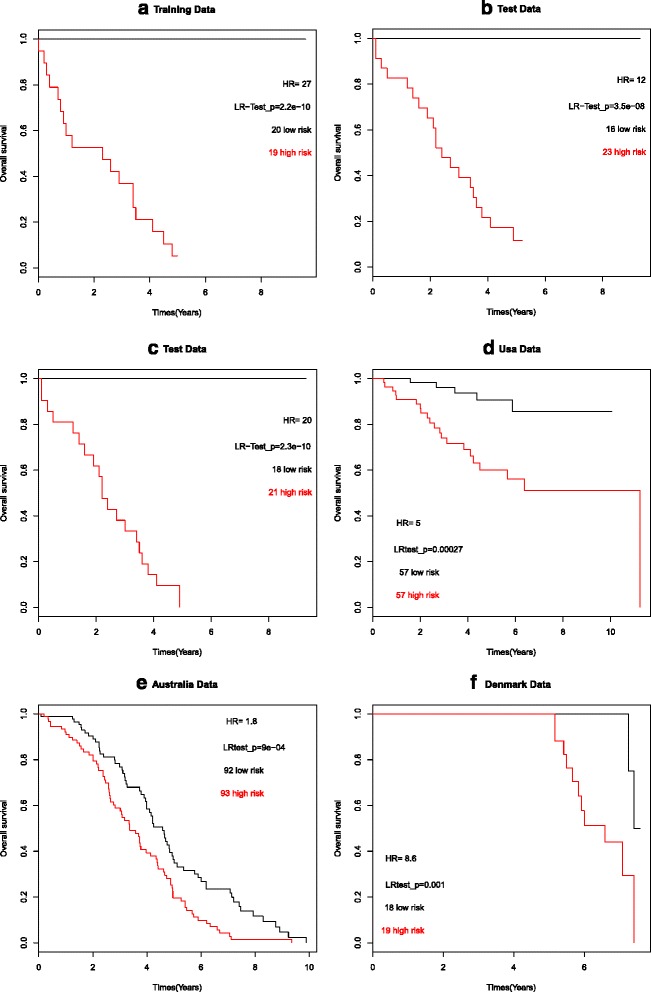
Survival analysis. Kaplan–Meier survival analysis using six different microarray datasets (Training and test sets (Illumina-based), dataset from Denmark (Affymetrix), dataset from the USA (Affymetrix) and dataset from Australia (Affymetrix)). The 19-gene signature segregates patients into two risk groups (red, high risk; black, low risk). The p values correspond to the likelihood ratio test comparing the survival curves

We performed the Elastic Net [23] to the training dataset and compute the risk scores using eq. (5) based on the model estimates to the training dataset and the test dataset. After calculating the risk score for each patient from the 19-gene expression signature as mentioned before, we divided the training set into high and low risk groups based on the cutoff value (-0.07) of the risk score. The likelihood ratio test was used to compare differences in overall survival between high and low risk groups in the training set 1 (likelihood ratio test, *p* <0.05; HR =27, (95 % CI, 5.165 – 140.5)) and test set 1 (likelihood ratio test, *p* <0.05, HR = 12, (95 % CI, 2.861 – 47.21)). Both Kaplan Meier survival plots (Fig. 3a and b) for training and test set 1 showed that this risk classification was significantly associated with the overall survival time. Similar results were observed in the other training and test sets. We also compared other two methods such as LASSO and Ridge regression with Elastic Net regression for prediction accuracy in our data. The prognostic index (risk score of 19 gene signature) was significantly associated with overall survival time in multivariate analysis (Table 3).

**Table 3:** Univariate and multivariate cox proportional hazard regression analyses

Genes	Univariate		Multivariate	
	Hazard ratio (95 % CI)	*p*-value	Hazard ratio (95 % CI)	*p*-value
*NOTCH2*	1.356 (1.154 – 1.592)	0.000 ***	1.56 (1.034 – 1.913)	0.009 **
*ITPRIP*	1.063 (0.919 – 1.23)	0.408	0.852 (0.614 – 1.183)	0.340
*FRMD6*	1.259 (1.092 – 1.452)	0.001 **	1.066 (0.850 – 1.338)	0.575
*GFRA4*	0.860 (0.728 – 1.016)	0.005 **	0.871 (0.547 – 1.005)	0.010 **
*OSBPL9*	0.803 (0.671 – 0.962)	0.017 **	0.818 (0.640 – 1.045)	0.047 **
*CPXCR1*	0.918 (0.811 – 1.041)	0.183’	1.030 (0.766 – 1.387)	0.840
*SORCS2*	1.159 (1.041 – 1.290)	0.006 **	0.936 (0.752 – 1.166)	0.558
*PDC*	0.812 (0.679 – 0.970)	0.022 **	1.068 (0.796 – 1.432)	0.659
*C12orf66*	0.902 (0.808 – 1.008)	0.069 *	1.007 (0.729 – 1.390)	0.965
*SLC38A9*	0.919 (0.845 – 1.000)	0.050 *	1.073 (0.894 – 1.289)	0.443
*OR10H5*	0.846 (0.704 – 1.018)	0.075 *	1.078 (0.826 – 1.407)	0.579
*TRIP13*	0.881 (0.802 – 0.968)	0.008 **	0.985 (0.780 – 1.244)	0.901
*MRPL52*	0.848 (0.767 – 0.939)	0.001 **	0.865 (0.697 – 1.075)	0.019 **
*DUSP21*	0.903 (0.807 – 1.011)	0.077 *	0.928 (0.697 – 1.235)	0.609
*BRCA1*	0.836 (0.764 – 0.915)	0.000 ***	0.884 (0.713 – 1.097)	0.264
*ELTD1*	1.155 (1.021 – 1.307)	0.021 **	0.969 (0.772 – 1.216)	0.787
*SPG7*	1.141 (1.035 – 1.259)	0.008 **	1.026 (0.840 – 1.255)	0.797
*LASS6*	0.867 (0.782 – 0.961)	0.006 **	0.788 (0.631 – 0.984)	0.035 **
*DUOX2*	1.076 (0.975 – 1.187)	0.141’	1.039 (0.844 – 1.280)	0.715
Age (>60)	0.154 (0.01953 – 1.225)	0.045 **	0.562 (0.0354 – 2.392)	0.049 **
Gender	1.048 (0.554 – 1.981)	0.886	1.729 (0.704 – 4.245)	0.231
Stage	1.644 (0.8567 – 3.156)	0.135’	1.274 (0.494 – 3.286)	0.615

This table shows the univariate and multivariate cox proportional hazard regression analyses of 19 gene signatures and other clinical variables associated with overall survival of CRC patients. [Relevant location: Page 14]

This study showed that the sensitivity and specificity of the 19-genes were 86.84 % and 87.50 % respectively which were acceptably higher than the ColoGuideEx with 72.22 % and 71.43 % respectively for 13-genes signature [14]. Meanwhile, ColoGuidePro [15] has the upmost value of analysis with 94.44 % and 90.48 % for 7-genes respectively. To identify genes that may predict overall survival, a univariate Cox proportion hazard regression analysis was performed with each of 19 differentially expressed genes in CRC in a cohort of 78 patients (Table 4). Table 4 shows that most of the genes including validated 5 genes (*FRMD6, MRPL52, TRIP13, ELTD1* and *SLC38A9*) were significantly correlated with the overall survival of the CRC patients. The five validated genes were significantly correlated with the overall survival with hazard ratios of 1.259 [*p* =0.001; 95 % confidence interval (CI): 1.092 to 1.452], 0.848 (*p* = 0.001; 95 % CI: 0.767 to 0.939), 0.881 (*p* = 0.008; 95 % CI: 0.802 to 0.968), 1.155 (*p* = 0.021; 95 % CI): 1.021 to 1.307) and 0.919 (*P* = 0.050; 95 % CI: 0.845 to 1) respectively.

**Table 4:** Comparison the LASSO and Ridge regression methods with Elastic Net regression

		Univariate		Multivariate	
		HR (95 % CI)	*P*	HR (95 % CI)	*P*
Datasets	Methods				
	Lasso	0.106 (0.030 – 0.370)	0.000	0.063 (0.016 – 0.252)	0.000
Our dataset	Ridge	0.812 (0.303 – 2.173)	0.000	0.083 (0.022 – 0.321)	0.000
	Elastic net	0.065 (0.014 – 0.287)	0.000	0.040 (0.008 – 0.198)	0.000
Denmark dataset	Lasso	0.055 (0.007 – 0.453)	0.007	0.044 (0.005 – 0.396)	0.005
	Ridge	0.112 (0.024 – 0.519)	0.005	0.080 (0.014 – 0.467)	0.005
	Elastic net	0.057 (0.007 – 0.464)	0.007	0.038 (0.004 – 0.389)	0.005
Australian dataset	Lasso	0.565 (0.396 – 0.805)	0.002	0.549 (0.384 – 0.784)	0.001
	Ridge	0.447 (0.312 – 0.641)	0.000	0.454 (0.316 – 0.651)	0.000
	Elastic net	0.523 (0.370 – 0.739)	0.000	0.529 (0.373 – 0.748)	0.000
USA dataset	Lasso	0.105 (0.010 – 1.068)	0.056	0.104 (0.010 – 1.052)	0.055
	Ridge	0.130 (0.013 – 1.294)	0.082	0.129 (0.013 – 1.283)	0.081
	Elastic net	0.120 (0. 012 – 1.195)	0.071	0. 122 (0. 012 – 1.214)	0. 072
Norway dataset	Lasso	---	---	---	---
	Ridge	---	---	---	---
	Elastic net	0.536 (0.300 – 0.957)	0.035	0.569 (0.318 – 1.018)	0.057

This table shows the comparison with the LASSO, Ridge regression and Elastic Net methods for 19 gene signatures based on our dataset and other external datasets from different countries. Univariate and multivariate Cox’s proportional hazard model analysis of prognostic factor (prognostic index or risk score) for overall survival

[Relevant location: Page 16]

To determine whether these 19 genes can be independent prognostic markers, multivariate analysis was also performed including other clinical parameters (age, gender and stage) as shown in Table 4. The results showed that five genes (*NOTCH2, GFRA4, OSBPL9*, *MRPL52* and *LASS6*) as independent predictors with hazard ratios of 1.56 (*P* = 0.009, 95 % CI: 1.034 to 1.913), 0.871 (*p* = 0.010, 95 % CI: 0.547 to 1.005), 0.818 (*p* = 0.047, 95 % CI: 0.640 to 1.045), 0.865 (*p* = 0.019, 95 % CI: 0.697 to 1.075) and 0.788 (*P* = 0.035, 95 % CI: 0.631 to 0.984) respectively. To improve the prognostic capability, a risk score was calculated based on the expression level of *NOTCH2, GFRA4*, *OSBPL9, MRPL52* and *LASS6* and corresponding regression coefficients. A patient’s risk score was calculated as the sum of the expression values of these genes. The results confirmed that the patients in the low-risk score group also had a better prognosis than those in the high-risk score group in the test set. This data suggest that the risk score based on these five genes can be used to stratify patients (Fig. 3c).

### Survival analysis of the 19-gene signature using the USA, Australia, Denmark and Norway datasets

We assessed the predictive power of the 19 gene signatures on the four cohort datasets from the USA (*n* = 114), Australia (*n* = 185), Denmark (*n* = 37) and Norway (*n* = 95). All the microarray data were obtained using the Affymetrix platform and we confirmed that the risk classification using the 19 genes were replicated in all three datasets. We found 17 out of 19 gene signatures were present in the datasets from the USA, Australia and Norway while 18 out of 19 genes were present in the dataset from Denmark.

The Kaplan Meier survival curves for the high and low risk score groups are shown in Fig. 3d-f. Patients with high risk scores showed significantly poorer overall survival than the patients with low risk scores for the USA dataset (likelihood ratio test *p*-value <0.01; HR = 4.9 (95 % CI, 1.827 – 12.99)), for the Australian dataset, (likelihood ratio test *p*-value <0.01; HR = 1.8 (95 % CI, 1.268 – 2.533)) and for the dataset from Denmark (likelihood ratio test *p*-value <0.01; HR = 8.6 (95 % CI, 1.842 – 40.05)). Interestingly, we observed that the median risk score for all external validation datasets as well as our training dataset situated between -0.099 and -0.038. We again compared LASSO and Ridge with Elastic Net regression for prediction patients into high and low risk survival groups in external datasets from different countries. The prognostic index was significantly associated with overall survival time in most of the external datasets in multivariate analysis (Table 3). We observed that the LASSO and Ridge methods fail to obtained prognostic index to measure the association, while the elastic net was significantly associated with overall survival time in the Norway dataset (Table 3).

### Validation of the microarray data

**Fig. 4:**
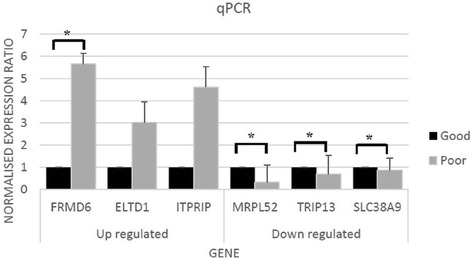
Validation of detected genes using qPCR. The normalized gene expression ratio for six genes including *FRMD6, ELTD1, ITPRIP*, *MRPL52, TRIP13* and *SLC38A9* which was determined using qPCR (*p* < 0.05). (*) represents the significant genes

Validation using qPCR demonstrated similar trends between poor and good survival groups when compared with the microarray data. All up-regulated genes (*FRMD6, ELTD1* and *ITPRIP*) and down-regulated genes (*MRPL52, TRIP13* and *SLC38A9*) were confirmed by qPCR according to 2^-∆∆C^_T_ method as seen in Fig. 4.

## Discussion

Microarray profiling allows the analysis of thousands of genes and the identification of differentially expressed genes which could then be used to characterize colorectal cancer from a molecular perspective. We performed a microarray study using the DASL assay on CRC patients with Dukes’ B and C to predict patient’s survival. This assay was designed with multiple probes per transcript to generate reproducible gene expression profiles from partially degraded RNA in archival tissues which had the advantage of information on the patients’ survival. The quality of microarray data for downstream analysis is important in order to answer correctly the research questions. Outlier detection in microarray data is desirable to avoid noise and statistical damage with the aim to minimize the risk of misinterpreting the biological events.

This study has successfully stratified colorectal cancer using a 19-gene signature and provides a molecular staging approach of patients into low risk and high risk groups. The final aim is to use this identifier in a personalized approach for CRC patients as there are weaknesses in using histopathological examination alone to prognosticate patients’ survival. The overall survival rate for CRC patients has increased, however, the individual survival rate for patients with Dukes’ B and C is still low. A previous study developed a molecular classifier based on a core set of 43 genes to predict the 3-year survival for patients [12]. The gene signatures were also validated on a different population using a different platform. In this study, we identified gene signatures which could predict the 5-year survival rate. This is important to allow the best plan of treatment to be given to patients while at the same time reducing unnecessary toxicity and aggressive side effects.

The Oncotype Dx and Coloprint for colorectal cancers are two different assays developed based on quantitative multi-gene RT PCR assay and oligonucleotide microarray respectively. Both assays were developed to improve risk stratification of relapse for patients with Stage II CRC. Oncotype Dx has a limitation in which identified genes were derived from four separate studies on individual genes and not determined using the whole genome microarray method. Therefore, the assay could probably miss important genes that may be involved in determining cancer relapse. Another disadvantage of the assay is the difficulty in assigning patients into groups of risk prediction due to the narrow range of prediction scores. These challenges hinder the effective use of this assay in clinical practice [25]. The gene expression data from Coloprint was not made publicly available hence the evaluation of the classifier cannot be performed [14]. A recent study developed 113 gene signatures from a published gene expression profile to predict prognostic risk [26]. Prognostic index for patients was calculated to discriminate patients into high- and low risk group. To show the prognostic significance, validation was done using independent data sets from different countries using the same platform. In this study, we completed the whole genome microarray and calculated a risk score for each patient. We used the median risk score as a cutoff point in which the median was not affected by outliers [27]. Using this median, the patients were efficiently separated into two groups i.e., high and low risk groups.

Previous clinical trials have used two robust gene classifiers called ColoGuide Ex and ColoGuide Pro. The investigators used the classifiers to stratify the prognosis of patients with stage II and III [14, 15]. The performances of both classifiers were validated using independent external datasets from different countries. The strength of the classifier is that it requires validation at the individual patient level conducted in the prospective trial. In our study, we revealed a set of genes which could provide a significant risk assessment approach for patients in both intermediate stages (Dukes’ B and C). Therefore, an inadequate sampling of lymph nodes as a risk factor in the conventional clinico-pathologic approach to determine staging could be disregarded [16, 28].

From our findings, some of the signature genes play roles in cell differentiation and amino acid transport as well as coding for phosphoproteins, receptors and membrane-associated proteins. We validated and confirmed six out of the 19 genes using RT-PCR. One of these is the EGF latrophilin and seven transmembrane domain-containing protein 1 (*ELTD1*) gene. A previous in vivo study found that a high expression (~3 fold increase, *p* <0.001) of this gene was associated with high grade gliomas and low survival rate [29]. In our study, *ELTDI* was consistently up-regulated in the group of CRC patients with poor survival. The *ELTDI* gene is a member of the secretin family of G protein–coupled peptide hormone receptors and belongs to the EGF-7 transmembrane subfamily. The EGF family plays important roles in cell division, apoptosis, differentiation and migration [30]. Wallgard et al. [31] reported that *ELDT1* is associated with microvasculature expression of endothelial cell-specific in vivo for tumor progression.

The second validated gene, thyroid hormone receptor interactor 13 (*TRIP13*), encodes a protein that cooperates with thyroid hormone receptors. High expression of *TRIP13* gene was reported to be associated with poor prognosis in breast cancer [32]. One of the gene in this panel was the sortilin-related VPS10 domain containing receptor 2 (*SORCS2)* which found to be up-regulated in the group with poor survival compared to those with good survival. This gene is normally highly expressed in the central nervous system during development [33, 34]. In contrast, this gene was documented to be down-regulated in the stromal cells of breast cancer and associated with poor outcome [35, 36]. Another one of the genes in the panel i.e., Notch homolog 2 (*NOTCH2*) has been widely reported to be linked with survival. NOTCH2 is a receptor for membrane bound ligands and has roles in vascular, renal and hepatic development [37, 38]. The *NOTCH2* gene was also reported to be an independent prognostic predictor of CRC [39]. A high expression of *NOTCH2* might predict good survival in CRC with a median survival of 45 months [39]. We noted an opposite effect of this gene in our current study. This is probably due to the heterogeneity of tissue samples with different stages of tumor tissues between the respective studies. Less information are available for six of the genes in the panel in relation to cancer namely the *ITPRIP, FRMD6, CPXCR1, SLC38A9, MRPL52* and *GFRA4*.

We showed that the performance of the 19-gene signature was reliable and also reproducible. This was evident through the use of four external validation series from other countries with different population settings. A similar study used information from other studies to validate the performance of their 5-gene panel [40]. Our study results show the robustness of the gene panel whereby the test successfully differentiate patients into groups with high and low risk of recurrence in the training, testing and also the external validation datasets.

## Conclusions

We have shown that our 19-gene signature is able to classify CRC patients into prognostic groups according to their risk scores. Patient who will be assigned into the high risk group could have a proper treatment plan, relevant chemotherapy dosage and effective medication strategy in order to increase the survival rate at the same time reduce the invasiveness of cancer cells. While for patients who were classified as the low risk group could avoid or received lower doses of adjuvant chemotherapy. Further validation tests are still required using larger number of samples. Future prospective clinical trials could also be conducted using this classifier to randomize treatment groups and explore further the sensitivity and specificity of this 19-gene signature.

## Additional file

Additional file 1: a) Detection of outliers. b) Summary of permutation analysis. a) Detection of outliers. Histogram of microarray data showing the presence of outliers after detection by using the beta weight function. Samples with weight index < 0.2 were considered as outliers. b) Summary of the permutation analysis for 78 CRC samples to identify differentially expressed genes. One hundred training and test set were generated and further analyzed using 3 statistical methods: SAM, LIMMA and *t*-test to calculate the p-value for each gene. (DOCX 452 kb)

## Abbreviations

cDNA: Complementary deoxyribonucleic acid
CRC: Colorectal cancer
DASL: cDNA-mediated annealing, selection, extension and ligation
DE: Differentially expressed
FFPE: Formalin-fixed paraffin embedded
GEO: Gene Expression Omnibus
LIMMA: Linear Model for Microarray Data
PCR: Polymerase chain reaction
rCI: Robust confidence interval
RMA: Robust Multichip Average
RNA: Ribonucleic acid
RT-PCR: Real-time polymerase chain reaction
SAM: Significant analysis of microarray
UKM: Universiti Kebangsaan Malaysia
USA: United States of America
