# Supplementary material for: A 19-Gene expression signature as a predictor of survival in colorectal cancer
Source: BMC Med Genomics. 2016 Sep 8;9(1):58. doi: 10.1186/s12920-016-0218-1 (PMC5016995; doi:10.1186/s12920-016-0218-1)
Supplement: Additional file 1: — a) Detection of outliers. b) Summary of permutation analysis. a) Detection of outliers. Histogram of microarray data showing the presence of outliers after detection by using the beta weight function. Samples with weight index < 0.2 were considered as outliers. b) Summary of the permutation analysis for 78 CRC samples to identify differentially expressed genes. One hundred training and test set were generated and further analyzed using 3 statistical methods: SAM, LIMMA and t-test to calculate the p-value for each gene. (DOCX 452 kb) [file 12920_2016_218_MOESM1_ESM.docx]

Supplementary Materials


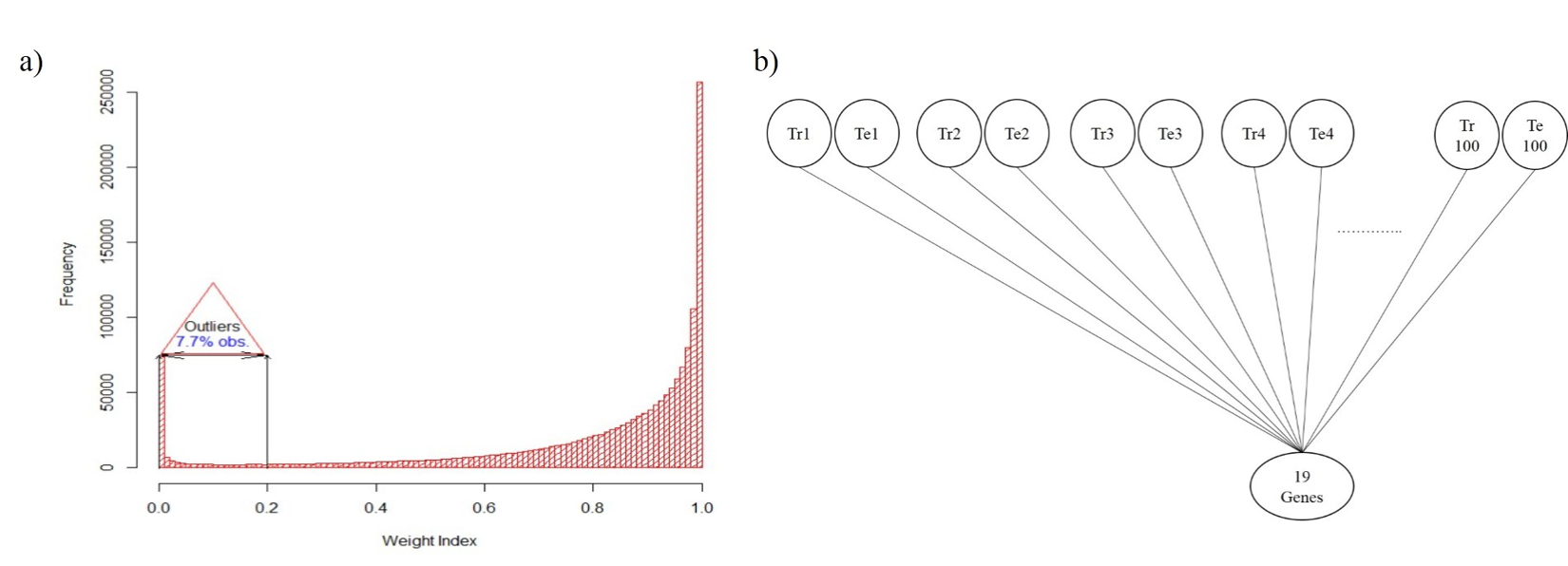


a) Detection of outliers. Histogram of microarray data showing the presence of outliers after detection by using the beta weight function. Samples with weight index < 0.2 were considered as outliers. b) Summary of the permutation analysis for 78 CRC samples to identify differentially expressed genes. One hundred training and test set were generated and further analyzed using 3 statistical methods: SAM, LIMMA and t-test to calculate the p-value for each gene.
